# Structural Causes of Pattern Formation and Loss Through Model-Independent Bifurcation Analysis

**DOI:** 10.21203/rs.3.rs-6098751/v1

**Published:** 2025-02-26

**Authors:** Liam D. O’Brien, Adriana T. Dawes

**Affiliations:** 1 Department of Mathematics, The Ohio State University, 231 W 18th Ave, Columbus, 43210, Ohio, USA.; 2 Department of Molecular Genetics, The Ohio State University, 484 W 12th Ave, Columbus, 43201, Ohio, USA.

**Keywords:** bifurcation theory, network dynamics, pattern formation, developmental biology, signaling networks, 34A34, 24C14, 34C23, 92C15, 92C40, 92C42

## Abstract

During development, precise cellular patterning is essential for the formation of functional tissues and organs. These patterns arise from conserved signaling networks that regulate communication both within and between cells. Here, we develop and present a model-independent ordinary differential equation (ODE) framework for analyzing pattern formation in a homogeneous cell array. In contrast to traditional approaches that focus on specific equations, our method relies solely on general assumptions about global intercellular communication (between cells) and qualitative properties of local intracellular biochemical signaling (within cells). Prior work has shown that global intercellular communication networks alone determine the possible emergent patterns in a generic system. We build on these results by demonstrating that additional constraints on the local intracellular signaling network lead to a single stable pattern which depends on the qualitative features of the network. Our framework enables the prediction of cell fate patterns with minimal modeling assumptions, and provides a powerful tool for inferring unknown interactions within signaling networks by analyzing tissue-level patterns.

## 1 Introduction

Pattern formation is a hallmark of developmental biology, where cells within a tissue or organism differentiate in a precise spatial arrangement to form complex structures. This process is essential for cell fate specification, tissue development, and morphogenesis; and understanding the molecular causes of pattern formation has promising applications in regenerative medicine, tissue engineering, and the treatment of congenital malformations (Chuong and Richardson, 2009).

During development, a group of cells with the same developmental potential will receive a signal called a *morphogen* that prompts them to communicate and form a pattern of cell types. These local patterning events occur sequentially, with each iteration creating progressively finer cellular patterns across the developing organism. Recent ordinary differential equation (ODE) models have considered one iteration, focusing on how changes in cell-communication and chemical kinetics affect the cellular pattern (Williamson et al., 2021; O’Dea and King, 2012; de Back et al., 2013; Fisher et al., 2005; Irons et al., 2010; Giurumescu et al., 2006; Vasilopoulos and Painter, 2016; Hadjivasiliou et al., 2016). Most models investigate the Notch signaling pathway, which is a key signaling pathway involved in contact-mediated cell-communication. Although the full complexity of the Notch pathway is difficult to model, simplified systems – such as the model from Collier et al. (1996) – have offered valuable insights into how cell-communication can give rise to fine-grained patterns.

Over time, modeling changes have produced further understanding. For instance, O’Dea and King (2012) incorporated morphogens that act as bifurcation parameters, showing how an initially homogeneous steady-state can be destabilized, leading to the formation of patterns. They also derived a continuum model to account for morphogen gradients, showing how tissues with different chemical gradients form different patterns. Williamson et al. (2021) used a similar approach and demonstrated that the structure of communication networks among cells determines the types of fine-grained patterns that can emerge. Others have shown via simulations that coarse-grained patterns such as spots and stripes can arise if there is long-range signaling in the Notch pathway (Vasilopoulos and Painter, 2016; Hadjivasiliou et al., 2016).

Despite the usefulness of these models, they all rely on specific equations; therefore, there are possible dynamics that the models cannot represent due to potentially incomplete assumptions about the system, including assumptions about reaction rates and the molecular pathways involved (e.g. focusing on Notch signaling). Moreover, although model investigation has helped with understanding which parameters are important for pattern formation, the inherent complexity of any model prohibits us from determining which parameters are necessary or sufficient for a pattern. Recent advances in network theory (Golubitsky and Stewart, 2023) allow a more thorough investigation of pattern formation in many cell-communication networks. By ignoring all unnecessary information and focusing on the connectivity of cells (i.e. which cells influence each other), one can predict all possible patterns of cell fates that can emerge in a generic system (Wang and Golubitsky, 2004). Additionally, we provide simple conditions – related to qualitative features of the chemical signaling network – that are both necessary and sufficient for a pattern of cell fates to form under our assumptions.

Using our theory, we find that despite the limited chemical signaling pathways involved in development, cells can generate diverse patterns by reorganizing themselves, thus validating and extending the work of Williamson et al. (2021), Vasilopoulos and Painter (2016), and Hadjivasiliou et al. (2016). On the other hand, cells whose organization is constrained can form various patterns by using different chemical signaling mechanisms, as suggested in de Back et al. (2013). We identify conditions under which cells will fail to form stable patterns, instead remaining in a homogeneous steady-state, oscillating synchronously, or forming oscillating patterns. Importantly, our theory shows that the chemical kinetics proposed in the Collier model are not the only kinetics that can lead to pattern formation. Finally, we are able to infer possible biochemical interactions from an observed pattern, which can reveal previously unknown interactions in biochemical signaling pathways.

The paper is organized as follows. In Section 2, we state our biological assumptions and introduce our mathematical framework, including the theory of network dynamics. We highlight how bifurcations in networks can give rise to patterns corresponding to eigenvectors of the system. In Section 3, we show that qualitative features of chemical signaling determine the stability of the initially homogeneous steady-state and dictate the critical eigenvectors that lead to pattern formation. In Section 3.2, we apply our theory to various biological contexts and predict patterns using minimal modeling assumptions. We show how the same chemical signaling pathway can produce different patterns depending on the cell-communication network. Finally, in Section 3.3, we demonstrate how our framework can be used to infer properties of biochemical signaling pathways from observed patterns in tissues.

## 2 Mathematical methods

### 2.1 General model assumptions

We make the following general assumptions to guide the construction of our network models and their associated internal dynamics.

1. The cells are well-mixed compartments that can be characterized by the concentration of multiple chemical species inside the cell.
2. The concentrations of chemical species within the cells change smoothly over time.
3. The cells begin in a nearly identical state.
4. The external signals to each cell are identical.
5. The cells are in a sufficiently uniform lattice structure. (The lattice may be in 1,2, or 3 spatial dimensions).
6. The cells average signals from their neighbors.

Assumptions (1) and (2) allow us to represent our system of cells with ordinary differential equations. Assumptions (3)-(6) allow us to represent our system with a *strongly connected, regular* network as defined in Section 2.

## 2.2 Network definitions and basic properties

Our approach emphasizes the role of network structure in pattern formation, so we outline key definitions and properties that will be used throughout the paper (for a fully rigorous treatment, see (Nijholt et al., 2016; Golubitsky and Stewart, 2023)).

**Definition 1** (Regular Networks). *A regular network is a finite directed graph* 𝒩=(𝒱,𝒜,s,t) *where*

1. 𝒱 *is the set of nodes*
2. 𝒜 *is the set of arrows*
3. s:𝒜→𝒱 *is the source map*
4. t:𝒜→𝒱 *is the target map*.

*Additionally, the number of input arrows to each node is the same (i.e*. $\left|t^{-1}\left(\left\{v_{1}\right\}\right)\right|=\left|t^{-1}\left(\left\{v_{2}\right\}\right)\right|$ *for all* $v_{1},v_{2}\in 𝒱$*). We call the number of input arrows the valence of the network*.

**Fig. 1:**
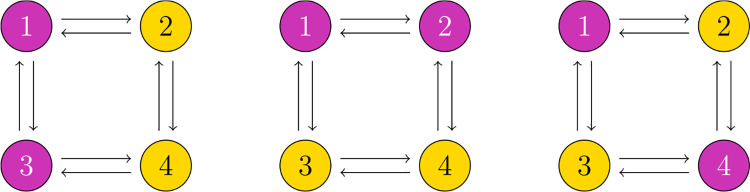
Each of these diagrams represents a balanced coloring on the regular four node network that is depicted. Indeed, in the left and center images, each yellow node gets one input from pink (dark) and one input from yellow (light), while both pink nodes get one input from pink and one from yellow. On the right, each pink node gets two inputs from yellow, and each yellow node gets two inputs from pink

For example, each network shown in Figure 1 (ignoring colors) is a regular network because every node has two input arrows.

There is a class of ODEs called *admissible* ODEs that is naturally associated with a regular network 𝒩. Suppose that each node v∈𝒱 has an associated “state” $x_{v}\in R^{s}$ – where $R^{s}$ is called the *node space*. Then the state of an n-node network with node space $R^{s}$ can be described by $x_{𝒩}={\left(x_{v}\right)}_{v\in V}\in R^{ns}$.

Next, suppose that each node v is only influenced by itself and nodes w such that there is an arrow from w to v (i.e. s(a)=w and t(a)=v for some a∈𝒜). Let ${\left\{v_{j}^{i}\right\}}_{1\leq j\leq \nu }$ denote the set of input nodes to $v_{i}$ (i.e. nodes satisfying $s(a)=v_{j}^{i}$ and $\left.t(a)=v_{i}\right)$.

**Definition 2** (Admissible ODE for a Regular Network). *An ODE is called admissible for the regular network* 𝒩 *if for some smooth function* f, *the dynamics of each node can be written in the form*

$${\dot{x}}_{v_{i}}=f\left(x_{v_{i}},\bar{x_{v_{1}^{i}},\ldots ,x_{v_{\nu }^{i}}}\right)$$

*where the overline indicates that* f *is symmetric in the arguments 2 through* ν+1.

**Definition 3** (Balanced Colorings of Regular Networks). *For a regular network* 𝒩=(𝒱,𝒜,s,t), *assign every vertex a color* {1,2,…,k} *via the coloring map*

κ:𝒱→{1,2,…,k}.

*This coloring is balanced if for every* $v_{1},v_{2}\in 𝒱$ *with* $\kappa \left(v_{1}\right)=\kappa \left(v_{2}\right)$ *there exists a color-preserving bijection between their input nodes*:

$$\alpha :\{s(a){\}}_{t(a)=v_{1}}\to \{s(a){\}}_{t(a)=v_{2}}.$$

Intuitively, a coloring is balanced if for any two nodes $v_{1},\;v_{2}$ with the same color, the set of nodes influencing $v_{1}$ has exactly the same colors, with the same frequency, as the set of nodes influencing $v_{2}$. See Figure 1 for examples of balanced colorings.

**Definition 4** (Polysynchrony Subspace). *Let* ⋈ *be a balanced coloring of a regular network* 𝒩=(𝒱,𝒜,s,t) *given by the coloring map* κ. *The associated polysynchrony subspace is the set*

$$\Delta_{⋈}=\left\{x_{𝒩}:x_{v_{1}}=x_{v_{2}}\;\text{whenever}\;\kappa \left(v_{1}\right)=\kappa \left(v_{2}\right)\right\}.$$

**Fig. 2:**
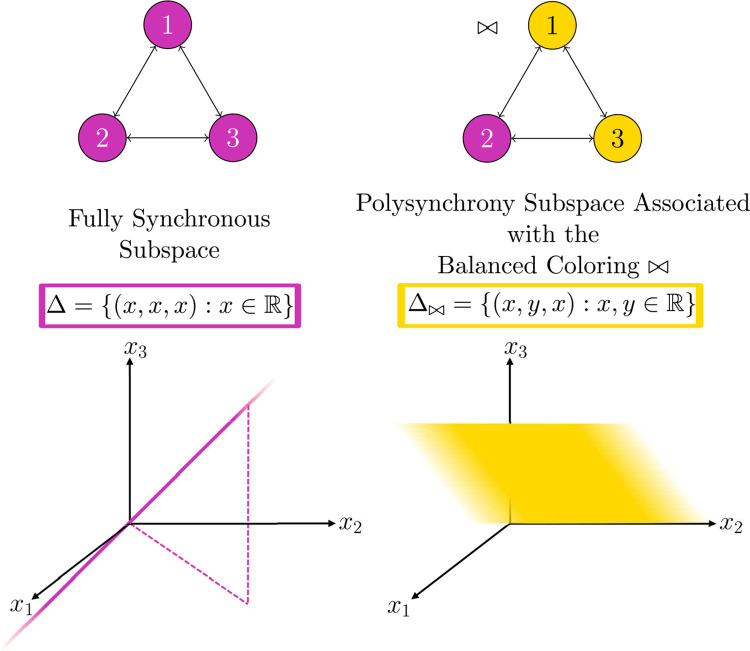
Classically, ODEs are imagined in phase space. Considering variables from a network perspective allows us to consider synchronous nodes, while maintaining a natural correspondence to phase space. If the node space is R, then the phase space of any admissible ODE for the depicted network is $R^{3}$. Balanced colorings represent patterns of synchrony that correspond to polysynchrony subspaces of the phase space. For example, if all cells are equal for all time, the trajectory of a solution to the ODE is inside the fully synchronous subspace Δ depicted on the bottom left. If $x_{1}(t)=x_{3}(t)$ for all time, we can represent the pattern of synchrony with a balanced coloring ⋈ in the network; and the coloring corresponds to the polysynchrony subspace $\Delta_{⋈}$ depicted on the bottom right

See Figures 1, 2 for examples of balanced coloring and corresponding polysynchrony subspaces.

**Definition 5** (Flow Invariance). *Let* F:X→X *be a smooth vector field. A subspace* E⊂X *is flow-invariant under* F *if* F(E)⊂E.

**Proposition 1** (Polysynchrony Subspaces are Flow-Invariant). *Let* ${\dot{x}}_{𝒩}=F\left(x_{𝒩}\right)$ *be an admissible ODE for the network* 𝒩. *Every polysynchrony subspace given by a balanced coloring is flow-invariant under* F.

The balanced colorings in Figure 1 from left to right correspond to the polysynchrony subspaces

$$\Delta_{1}=\left\{(x,y,x,y)\in R^{4s}:\;x,y\in R^{s}\right\}$$

$$\Delta_{2}=\left\{(x,x,y,y)\in R^{4s}:\;x,y\in R^{s}\right\}$$

$$\begin{array}{c} \Delta_{3}=\left\{(x,y,y,x)\in R^{4s}:\;x,y\in R^{s}\right\} \end{array}.$$

Coloring every node the same is always balanced, and the associated polysynchrony subspace is called the *fully synchronous subspace*, which we denote Δ. If $x_{𝒩}\in \Delta$, we say that the system is *fully synchronous*.

### 2.3 Eigenvalues depend on cell-level dynamics and network structure

Consistent with our general assumptions discussed above, we assume that any admissible ODE of a regular network with n nodes takes the form

$$\begin{array}{ll} {\dot{x}}_{v_{1}} & =f\left(x_{v_{1}},\bar{x_{v_{1}^{1}},\ldots ,x_{v_{\nu }^{1}}},\lambda \right)\\ & \vdots \\ {\dot{x}}_{v_{n}} & =f\left(x_{v_{n}},\bar{x_{v_{1}^{n}},\ldots ,x_{v_{\nu }^{n}}},\lambda \right) \end{array}$$

where λ∈R is a bifurcation parameter.

Let $x={\left(x_{v_{i}}\right)}_{1\leq i\leq n}$ denote the total state of the network, and suppose x∈Δ. If we write the arguments of f as

$$f≔f\left(u,\bar{v_{1},\ldots ,v_{\nu }},\lambda \right)$$

then the *internal and coupled dynamics* of the system (respectively) are given by

$$Q(x,\lambda )≔{\left.D_{u}f\right|}_{\left(x,\lambda \right)}$$

$$R(x,\lambda )≔{\left.D_{v_{1}}f\right|}_{\left(x,\lambda \right)}\left(={\left.D_{v_{2}}f\right|}_{\left(x,\lambda \right)}=\ldots ={\left.D_{v_{\nu }}f\right|}_{\left(x,\lambda \right)}\right)$$

where $D_{y}f$ represents the differential of f with respect to the variable $y\in R^{s}$. For example,

$$D_{y}f=\left(\begin{array}{cccc} \frac{\partial f_{1}}{\partial y_{1}} & \frac{\partial f_{1}}{\partial y_{2}} & \cdots & \frac{\partial f_{1}}{\partial y_{s}}\\ \frac{\partial f_{2}}{\partial y_{1}} & \frac{\partial f_{2}}{\partial y_{2}} & \cdots & \frac{\partial f_{2}}{\partial y_{s}}\\ \vdots & \vdots & \ddots & \vdots \\ \frac{\partial f_{s}}{\partial y_{1}} & \frac{\partial f_{s}}{\partial y_{2}} & \cdots & \frac{\partial f_{s}}{\partial y_{s}} \end{array}\right).$$

We remove the arguments (x,λ) of Q and R if it is clear where they are being evaluated.

**Definition 6** (Adjacency Matrix). *The adjacency matrix of a regular network* 𝒩=(𝒱,𝒜,s,t) *with n nodes is a* n×n *matrix* $A=\left(a_{ij}\right)$ *with entries* $a_{ij}$ *defined to be the number of arrows from node* $v_{j}$ *to node* $v_{i}$ $(i.e.\;\left.\left|\left\{a\in 𝒜:s(a)=v_{j},t(a)=v_{i}\right\}\right|\right)$.

**Proposition 2** (Eigenvalues of System via Reduced Matrices). *Suppose* 𝒩 *is a regular network with adjacency matrix* A *and admissible* $ODE\dot{x}=F(x,\lambda )$. *If* $\mu_{1},\ldots ,\mu_{k}$ *are the eigenvalues of* A *(not necessarily distinct) with eigenvectors* $v_{1},\ldots ,v_{k}$, *and* $J=(dF{)}_{\left(x,\lambda \right)}$ *is the Jacobian of* F *at a synchronous equilibrium* (x,λ)∈Δ×R, *then the eigenvalues of J are the union of the eigenvalues of* $Q+\mu_{j}R$ *where* Q *and* R *are the internal and coupled dynamics, respectively. Furthermore, the corresponding eigenvectors are* $u⊗v_{j}$ *where* u *is an eigenvector of* $Q+\mu_{j}R$.

*Proof*. See (Golubitsky and Stewart, 2023).

### 2.4 The critical eigenvalue dictates the preferred pattern

We assume that pattern formation occurs from a synchrony-breaking bifurcation and argue the first bifurcating pattern is the “preferred” pattern of a tissue. When the homogeneous steady-state is destabilized via a bifurcation, the critical eigenvalue corresponds to a pattern (Theorem 3), which is generically the only stable bifurcating pattern (Proposition 4). We assume that the bifurcation parameter λ changes much slower than the state of the cells. With a quasistatically (slowly) varying λ, the uniform state can lose stability with a stable patterned state emerging; thus, the system will move from the homogeneous state to the pattern state and will remain there unless there is a secondary bifurcation on the pattern branch. Furthermore, as in Poston and Stewart (1978), we may assume that the only bifurcations present are those that are forced by our assumptions and observed data. Extraneous secondary bifurcations would violate Occam’s Razor – the principle that the simplest model which can explain observed phenomena is best. Even with secondary bifurcations, the preferred pattern is the only stable pattern for some region of the parameter space, and it is reasonable that unusual behaviors can arise for unrealistic parameter values.

**Theorem 3** (Patterns Bifurcating from Synchrony). *Let* 𝒩 *be a regular network with admissible* $ODE\dot{x}=F(x,\lambda )$. *Let* Δ *denote the fully synchronous subspace, and let* $\Delta_{⋈}\neq \Delta$ *be the polysynchrony subspace associated with the balanced coloring* ⋈. *Assume that there is a synchronous equilibrium* $\left(x_{0},\lambda_{0}\right)$ *and that* $J≔(dF{)}_{\left(x_{0},\lambda_{0}\right)}$ *has a critical eigenvalue* μ. *Denote the associated generalized eigenspace* $E^{\mu }$. *If* $E^{\mu }\cap \Delta =\{0\}$ and $\text{dim}\left(E^{\mu }\cap \Delta_{⋈}\right)=1$, *then generically a unique branch of equilibria with synchrony pattern* ⋈ *bifurcates from* $x_{0}$ *at* $\lambda_{0}$.

*Proof (modified from* (Golubitsky and Stewart, 2023)). Since $E^{\mu }\cap \Delta =\{0\},{\left.J\right|}_{\Delta }$ is nonsingular. Thus, by the implicit function theorem, there exists a synchronous branch of equilibria in the neighborhood of $\left(x_{0},\lambda_{0}\right)$. By a change of coordinates, we can assume that the synchronous branch is given by F(0,λ)=0. Since $\text{dim}\left(E^{\mu }\cap \Delta_{⋈}\right)=1$, the kernel of ${\left.J\right|}_{\Delta_{⋈}}$ is 1-dimensional, so we can use Liapunov-Schmidt reduction to find a reduced equation of the restriction ${\left.F\right|}_{\Delta_{⋈}}:\Delta_{⋈}\times R\to \Delta_{⋈}$ given by

$$g:{\text{span}}_{R}\left\{v\right\}\times R\to {\text{span}}_{R}\left\{v^{*}\right\}.$$

for some vectors $v,\;v^{*}$. The zeros of g are in one-to-one correspondence with the zeros of ${\left.F\right|}_{\Delta_{⋈}}$ in a neighborhood of the bifurcation.

Since the spatial domain and codomain of g are 1-dimensional, we can write

$$g(sv,\lambda )=h(s,\lambda )v^{*}$$

for s∈R and h:R×R→R. Liapunov-Schmidt can be chosen to preserve the existence of the trivial solution, so we may assume h(0,λ)=0.

The eigenvalue crossing condition and reduction imply that $h_{\lambda }(0,0)$ is generically nonzero. Thus, by the implicit function theorem h(s(λ),λ))=0 for some function s of λ. Since we can write s as a function of λ in a neighborhood of the bifurcation, there exists a branch of solutions to g=0 and thus to ${\left.F\right|}_{\Delta_{⋈}}=0$. □

**Proposition 4** (Subsequent Branches are Unstable). *Suppose* 𝒩 *is a regular network with admissible* $ODE\dot{x}=F(x,\lambda )$. *Let* A *be the adjacency matrix with eigenvalues* $\mu_{1},\ldots ,\mu_{k}$, *and assume there exists a synchronous branch of equilibria* (x(λ),λ)∈Δ×R *that is stable for* $\lambda \in \left(\lambda_{0}-\delta ,\lambda_{0}\right)$ *(for some* δ>0*)*. *Suppose that* $(dF{)}_{\left(x\left(\lambda_{0}\right),\lambda_{0}\right)}$ *has a critical eigenvalue that is an eigenvalue of the matrix* $Q+\mu_{\ell }R$. *Furthermore, suppose that for* $\lambda_{0}<\lambda \leq \lambda_{1},(dF{)}_{\left(x(\lambda ),\lambda \right)}$ *has no critical eigenvalues that are eigenvalues of* $Q+\mu_{\ell }R$. *If there is a bifurcation at* $\left(x_{0}\left(\lambda_{1}\right),\lambda_{1}\right)$ *with critical eigenvalue from* $Q+\mu_{s}R$ *for* s≠ℓ, *then generically the bifurcating branches are unstable*.

*Proof*. We initially assume that the synchronous equilibrium is stable. If there is a bifurcation at $(\left.x\left(\lambda_{0}\right),\lambda_{0}\right)$ with a critical eigenvalue of $Q+\mu_{\ell }R$, the synchronous equilibrium will generically lose stability. In particular, there will be a positive eigenvalue ξ(x(λ),λ) that is an eigenvalue of $Q(x(\lambda ),\lambda )+\mu_{\ell }R(x(\lambda ),\lambda )$. If there are no critical eigenvalues of $Q+\mu_{\ell }R$ for $\lambda_{0}<\lambda \leq \lambda_{1}$, then ξ(x(λ),λ) will remain positive at the bifurcation point $\left(x\left(\lambda_{1}\right),\lambda_{1}\right)$. Since the eigenvalues of the Jacobian $(dF{)}_{\left(x,\lambda \right)}$ continuously depend on (x,λ), the bifurcating branches will be unstable in some neighborhood of the bifurcation point. □

### 2.5 Other preliminaries

For our results, we will use the definition of a strongly connected graph and the well-known Perron-Frobenius theorem.

**Definition 7** (Strongly Connected Network). *A network* 𝒩 *is called strongly connected if for every pair of nodes* $\left\{v_{i},v_{j}\right\}$ *with* i≠j, *there is a path from* $v_{i}$ *to* $v_{j}$ *and from* $v_{j}$ *to* $v_{i}$.

**Theorem 5** (Perron-Frobenius). *If a network* 𝒩 *is strongly connected, then the adjacency matrix* A *has a simple positive eigenvalue* μ *equal to the spectral radius* ρ(A). *For all other eigenvalues* η *of A*, |η|<ρ(A).

*Proof*. See (Golubitsky and Stewart, 2023).

## 3 Results

### 3.1 The internal and coupled dynamics determine the preferred pattern

The adjacency matrix of the cell-communication network determines the possible patterns that can form in the tissue. Qualitative features of the internal and coupled dynamics select the preferred pattern from all possible ones.

We assume that 𝒩=(𝒱,𝒜,s,t) is a strongly connected regular network on n nodes without self-arrows (i.e. for any v∈𝒱 there is no a∈𝒜 with s(a)=v and t(a)=v). Denote the adjacency matrix A. We consider an arbitrary admissible ODE $\dot{x}=F(x,\lambda )$ with a synchronous branch of equilibria (x(λ),λ)∈Δ×R that is stable for $\lambda <\lambda_{0}$. We assume that the Jacobian of the ODE is singular at $\left(x_{0},\lambda_{0}\right)≔\left(x\left(\lambda_{0}\right),\lambda_{0}\right)$. Lastly, we take Q(x,λ) and R(x,λ) to be the linearized internal and coupled dynamics (respectively), which are defined whenever (x,λ)∈Δ×R (Section 2.3).

**Definition 8** (Critical Pattern Space). *Suppose that* A *has distinct eigenvalues* $\mu_{1},\mu_{2},\ldots ,\mu_{k}$ *and corresponding eigenvectors* ${\left\{v_{\alpha_{1}}\right\}}_{\alpha_{1}\in {𝒜}_{1}},{\left\{v_{\alpha_{2}}\right\}}_{\alpha_{2}\in {𝒜}_{2}},\ldots ,{\left\{v_{\alpha_{k}}\right\}}_{\alpha_{k}\in {𝒜}_{k}}$. *Let* $P^{\mu_{j}}$ *be given by*

$$P^{\mu_{j}}≔{\text{span}}_{R}{\left\{v_{\alpha_{j}}\right\}}_{\alpha_{j}\in {𝒜}_{j}}.$$

*Suppose that* $(dF{)}_{\left(x_{0},\lambda_{0}\right)}$ *has critical eigenvalues that by Proposition 2 are null eigenvalues of* ${\left\{Q+\mu_{j}R\right\}}_{j\in 𝒥}$ *for some* 𝒥⊂{1,2,…,k}. *Then define the critical pattern space to be the subspace of* $R^{n}$ *given by*

$$\underset{j\in J}{⨁}\;P^{\mu_{j}}.$$

**Proposition 6** (Stability of Synchronous 1-Dimensional Nodes). *If the node space is 1-dimensional and the synchronous equilibrium* (x(λ),λ) *is stable, then* Q(x(λ),λ)<0.

**Lemma 6.1.** The adjacency matrix A always has a strictly positive eigenvalue and an eigenvalue with strictly negative real part.

*Proof of lemma*. The adjacency matrix of a regular graph always has a unique largest eigenvalue equal to its valence $\mu_{k}>0$ (see Definition 1). Furthermore, since we assume our graph has no self-arrows, every element on the diagonal of A is zero, and tr(A)=0. Since the trace is the sum of the eigenvalues and there exists a positive eigenvalue, there must be some eigenvalues with negative real part. In particular, the eigenvalue with smallest real part, which we denote $\mu_{1}$, has negative real part. □

*Proof of proposition*. Recall that the eigenvalues of the system are $Q+\mu_{i}R$ where $\mu_{i}$ are the eigenvalues of the adjacency matrix A (Proposition 2). By Lemma 6.1, A has a positive eigenvalue $\mu_{k}$ and an eigenvalue $\mu_{1}$ with negative real part. Let each eigenvalue be written

$$\mu_{j}=u_{j}+\iota v_{j}$$

where $\iota =\sqrt{-1}$. For the remainder of this proof, we will drop the arguments of the internal and coupled dynamics, Q and R (respectively), writing

Q≔Q(x(λ),λ)

R≔R(x(λ),λ)

Since (x(λ),λ) is stable, the real part of each eigenvalue $Q+u_{i}R<0$ for all i. By Lemma 6.1, we can choose $\mu_{j}$ such that $\text{sgn}\left(u_{j}\right)=\text{sgn}(R)$. Then

$$Q+u_{j}R$$

$$=Q+\left|u_{j}R\right|<0$$

$$\Rightarrow Q<-\left|u_{j}R\right|\leq 0.$$

□

**Proposition 7** (Stability of Synchronous 2-dimensional Nodes). Let the node space be 2-dimensional, and suppose that A has distinct real eigenvalues $\mu_{1}<\mu_{2}<\ldots <\mu_{k}$, not counting multiplicity. If the synchronous branch (x(λ),λ) is stable, then tr(Q(x(λ),λ))<0.

*Proof.* Since (x(λ),λ) is stable,

$$\text{det}\left(Q+\mu_{i}R\right)>0$$

$$\text{tr}\left(Q+\mu_{i}R\right)<0$$

for 1≤i≤k. By Lemma 6.1, there exists positive and negative eigenvalues of A. Let $\mu_{j}$ be an eigenvalue with the same sign as tr(R). Then

$$\text{tr}\left(Q+\mu_{j}R\right)=\text{tr}\left(Q\right)+\mu_{j}\text{tr}\left(R\right)\;=\text{tr}\left(Q\right)+\left|\mu_{j}\text{tr}\left(R\right)\right|<0\;\Rightarrow \text{tr}\left(Q\right)<-\left|\mu_{j}\text{tr}\left(R\right)\right|\leq 0.$$

□

**Lemma 7.1.** *Suppose that* p(μ,λ):R×R→R *is a polynomial in* μ *smoothly parameterized by* λ. *Let* $\mu_{1}<\mu_{2}<\ldots <\mu_{k}$ *be a set of points in* R. *Let* δ>0, *and suppose that for all* $\lambda \in \left(\lambda_{0}-\delta ,\lambda_{0}\right)$ *we have* $p\left(\mu_{i},\lambda \right)<0$ *for all* i. *Suppose that at* $\lambda_{0},\;p\left(\mu ,\lambda_{0}\right)=\alpha \left(\lambda_{0}\right)+\beta \left(\lambda_{0}\right)\mu$ *is linear in* μ *and there exists some* j *such that* $p\left(\mu_{j},\lambda_{0}\right)=0$. *Then we have the following cases*

1. $p\left(\mu_{i},\lambda_{0}\right)=0$ *for all* i *if and only if* $\beta \left(\lambda_{0}\right)=0$.
2. $p\left(\mu_{1},\lambda_{0}\right)=0$ *and* $p\left(\mu_{i},\lambda_{0}\right)<0\forall i\neq 1$ *if and only if* $\beta \left(\lambda_{0}\right)<0$.
3. $p\left(\mu_{k},\lambda_{0}\right)=0$ *and* $p\left(\mu_{i},\lambda_{0}\right)<0\forall i\neq k$ *if and only if* $\beta \left(\lambda_{0}\right)>0$.

*Proof*. For the forward direction of (1), if $p\left(\mu_{i},\lambda_{0}\right)=0$ for all i, then $p\left(\cdot ,\lambda_{0}\right)\equiv 0$ since it is a linear function of μ; therefore, $\beta \left(\lambda_{0}\right)=0$. For the converse, suppose that $\beta \left(\lambda_{0}\right)=0$. Then $p\left(\mu ,\lambda_{0}\right)=\alpha \left(\lambda_{0}\right)$. By hypothesis, there exists some $\mu_{j}$ such that $p\left(\mu_{j},\lambda_{0}\right)=\alpha \left(\lambda_{0}\right)=0$. Clearly, this implies that $\alpha \left(\lambda_{0}\right)=0$. Thus, $p\left(\cdot ,\lambda_{0}\right)\equiv 0$, so $p\left(\mu_{i},\lambda_{0}\right)=0$ for all i.

For the forward direction of (2), suppose that $p\left(\mu_{1},\lambda_{0}\right)=0$ and $p\left(\mu_{i},\lambda_{0}\right)<0$ for all i≠1. This implies that for any i≠1,

$$\alpha \left(\lambda_{0}\right)+\beta \left(\lambda_{0}\right)\mu_{i}<\alpha \left(\lambda_{0}\right)+\beta \left(\lambda_{0}\right)\mu_{1}.$$

Equivalently,

$$\beta \left(\lambda_{0}\right)\mu_{i}<\beta \left(\lambda_{0}\right)\mu_{1}.$$

Since $\mu_{1}<\mu_{i}$, we must have that $\beta \left(\lambda_{0}\right)<0$.

For the converse, suppose that $\beta \left(\lambda_{0}\right)<0$. If

$$p\left(\mu_{j},\lambda_{0}\right)=\alpha \left(\lambda_{0}\right)+\beta \left(\lambda_{0}\right)\mu_{j}=0\;\text{for}\;\text{some}\;j\neq 1,$$

then since $\mu_{1}<\mu_{j}$,

$$p\left(\mu_{1},\lambda_{0}\right)=\alpha \left(\lambda_{0}\right)+\beta \left(\lambda_{0}\right)\mu_{1}>0,$$

but this contradicts the continuity of p(μ,λ) with respect to λ since $p\left(\mu_{1},\lambda \right)<0$ for $\lambda \in \left(\lambda_{0}-\delta ,\lambda_{0}\right)$. Hence, $\mu_{1}$ must be the unique point from $\left\{\mu_{1},\ldots ,\mu_{k}\right\}$ with $p\left(\mu_{1},\lambda_{0}\right)=0$.

For the forward direction of (3), suppose that $p\left(\mu_{k},\lambda_{0}\right)=0$ and $p\left(\mu_{i},\lambda_{0}\right)<0$ for all i≠k. This implies that for any i≠k,

$$\alpha \left(\lambda_{0}\right)+\beta \left(\lambda_{0}\right)\mu_{i}<\alpha \left(\lambda_{0}\right)+\beta \left(\lambda_{0}\right)\mu_{k}.$$

Equivalently,

$$\beta \left(\lambda_{0}\right)\mu_{i}<\beta \left(\lambda_{0}\right)\mu_{k}.$$

Since $\mu_{i}<\mu_{k}$, we must have that $\beta \left(\lambda_{0}\right)>0$.

For the converse, suppose that $\beta \left(\lambda_{0}\right)>0$. If

$$p\left(\mu_{j},\lambda_{0}\right)=\alpha \left(\lambda_{0}\right)+\beta \left(\lambda_{0}\right)\mu_{j}=0\;\text{for}\;\text{some}\;j\neq k,$$

then since $\mu_{j}<\mu_{k}$,

$$p\left(\mu_{1},\lambda_{0}\right)=\alpha \left(\lambda_{0}\right)+\beta \left(\lambda_{0}\right)\mu_{k}>0,$$

but this contradicts the continuity of p(μ,λ) with respect to λ since $p\left(\mu_{k},\lambda \right)<0$ for $\lambda \in \left(\lambda_{0}-\delta ,\lambda_{0}\right)$. Hence, $\mu_{k}$ must be the unique point from $\left\{\mu_{1},\ldots ,\mu_{k}\right\}$ with $p\left(\mu_{k},\lambda_{0}\right)=0$. □

**Theorem 8** (Characterizing the First Branch in 1-Dimension). *Let* A have eigenvalues $\mu_{1},\ldots ,\mu_{k}$ *ordered by real part, and assume that the node space is 1-dimensional. Then we have the following mutually exclusive cases*

1. *The critical pattern space is* $R^{n}$ *if and only if* $R\left(x_{0},\lambda_{0}\right)=0$.
2. *The critical pattern space is* $P^{\mu_{1}}$, *and the bifurcation is synchrony-breaking if and only if* $R\left(x_{0},\lambda_{0}\right)<0$.
3. *The critical pattern space is* $P^{\mu_{k}}={\text{span}}_{R}\left\{{\vec{1}}_{n}\right\}$ *(a vector of ones of length* n), *and the bifurcation is synchrony-preserving if and only if* $R\left(x_{0},\lambda_{0}\right)>0$.

*Proof*. *Let* $\mu_{1},\mu_{2},\ldots ,\mu_{k}$ be the eigenvalues of A with distinct real parts $u_{1}<u_{2}<\ldots <u_{\ell }$. Take p(u,λ) to be the linear polynomial in u given by

p(u,λ)=Q(x(λ),λ)+R(x(λ),λ)u,

so that $p\left(u_{i},\lambda \right)$ gives the real part of some eigenvalue of J. Since we assume that the synchronous branch is stable before the bifurcation, there exists δ>0 such that for all $\lambda \in \left(\lambda_{0}-\delta ,\lambda_{0}\right),p\left(u_{i},\lambda \right)<0$ for all i. Furthermore, at the bifurcation point, there exists some $u_{j}$ with $p\left(u_{j},\lambda_{0}\right)=0$. Therefore, the hypotheses of Lemma 7.1 are satisfied.

Applying Lemma 7.1, we have the following three cases.

1. $p\left(u_{i},\lambda_{0}\right)=0$ for all i if and only if $R\left(x\left(\lambda_{0}\right),\lambda_{0}\right)=0$.
2. $p\left(u_{1},\lambda_{0}\right)=0$ and $p\left(u_{i},\lambda_{0}\right)<0\forall i\neq 1$ if and only if $R\left(x\left(\lambda_{0}\right),\lambda_{0}\right)<0$.
3. $p\left(u_{k},\lambda_{0}\right)=0$ and $p\left(u_{i},\lambda_{0}\right)<0\forall i\neq k$ if and only if $R\left(x\left(\lambda_{0}\right),\lambda_{0}\right)>0$.

$p\left(u_{j}\right)=0$ exactly when the system has the critical eigenvalue $Q+\mu_{j}R$. Using Definition 8 the main conclusion immediately follows.

Lastly, the unique eigenvalue of A with largest real part $\mu_{k}$ is the unique eigenvalue with eigenvector $v_{k}=\vec{1}$ (a vector of ones). By Proposition 2 and Theorem 3, the bifurcation with critical eigenvalue $Q+\mu_{k}R$ is synchrony-preserving, whereas bifurcations with other critical eigenvalues are generically synchrony-breaking. □

**Theorem 9** (Characterization of Bifurcations in 2-dimensions with det(R)=0). *Suppose that the node space is 2-dimensional and the adjacency matrix* A *has real eigenvalues* $\mu_{1}<\mu_{2}<\ldots <\mu_{k}$, *where each* $\mu_{i}$ *has algebraic multiplicity* $\alpha_{i}$ *and geometric multiplicity* $\beta_{i}$. *Suppose that* $\text{det}\left(R\left(x_{0},\lambda_{0}\right)\right)=0$, *and take*

$$B\left(x_{0},\lambda_{0}\right)≔\text{tr}\left(Q\left(x_{0},\lambda_{0}\right)\right)\text{tr}\left(R\left(x_{0},\lambda_{0}\right)\right)-\text{tr}\left(Q\left(x_{0},\lambda_{0}\right)R\left(x_{0},\lambda_{0}\right)\right).$$

*In* Table 1, *we enumerate all possible critical pattern spaces, the multiplicity and type of critical eigenvalues, and the dimension of the critical eigenspaces. For the first four cases listed, we give necessary and sufficient conditions on the internal and coupled dynamics for such a bifurcation to occur. For the remaining cases, we give necessary conditions, which are sufficient if we assume the bifurcation is sufficiently degenerate*.

*Proof*. We want to use the trace and determinant to obtain information about the eigenvalues; therefore, define the following polynomials of μ:

$$p_{1}\left(\mu ,\lambda \right)=\text{tr}\left(Q\left(x\left(\lambda \right),\lambda \right)+\mu R\left(x\left(\lambda \right),\lambda \right)\right)\;=\text{tr}(\left(Q\left(x\left(\lambda \right),\lambda \right)\right)+\mu \text{tr}\left(R\left(x\left(\lambda \right),\lambda \right)\right)\;p_{2}\left(\mu ,\lambda \right)=\text{det}\left(Q\left(x\left(\lambda \right),\lambda \right)+\mu R\left(x\left(\lambda \right),\lambda \right)\right)$$

We will drop the argument λ if it is obvious where the polynomial is being evaluated. Since we assume that $\text{det}\left(R\left(x_{0},\lambda_{0}\right)\right)=0$, we have that

$$p_{2}\left(\mu ,\lambda_{0}\right)=[\text{tr}\left(Q\left(x_{0},\lambda_{0}\right)\text{tr}\left(R\left(x,\lambda \right)\right)-\text{tr}\left(Q\left(x_{0},\lambda_{0}\right)R\left(x_{0},\lambda_{0}\right)\right)\right]\mu +\text{det}\left(Q\left(x_{0},\lambda_{0}\right)\right)\;=\text{det}\left(Q\left(x_{0},\lambda_{0}\right)\right)+\mu B\left(x_{0},\lambda_{0}\right)$$

**Fig. 3:**
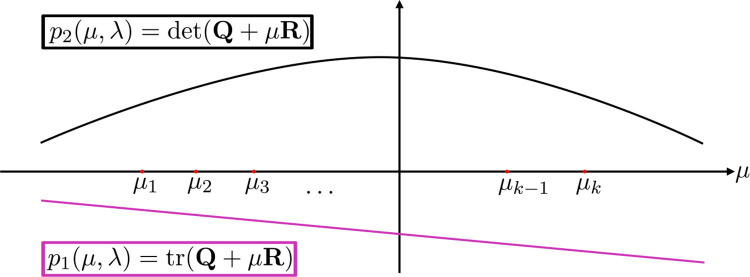
Illustration of the proofs for Theorems 9–12. Consider the trace and determinant of Q+μR as polynomials in μ with A’s eigenvalues $\mu_{1},\ldots ,\mu_{k}$ being points in the domain. By stability assumptions, $p_{2}\left(\mu_{i}\right)>0$ and $p_{1}\left(\mu_{i}\right)<0$ for all i when $\lambda <\lambda_{0}$. If $p_{1}$ or $p_{2}$ has a root that is some $\mu_{i}$ a bifurcation may occur. The trace is linear in μ, and when det(R)=0, the determinant is linear. Assuming that one of the graphs intersects $\left(\mu_{i},0\right)$ for some i, we can determine those i satisfying $p_{1}\left(\mu_{i}\right)=0$ or $p_{2}\left(\mu_{i}\right)=0$ from the slope of each line

We also assume the synchronous equilibrium is initially stable, so there exists δ>0 such that for all $\lambda \in \left(\lambda_{0}-\delta ,\lambda_{0}\right)$,

(1) $$p_{1}\left(\mu_{i}\right)<0$$

(2) $$\begin{array}{c} p_{2}\left(\mu_{i}\right)>0 \end{array}$$

for all i. This setup is illustrated in Figure 3. For a bifurcation to happen, there exists $\mu_{j}$ such that either $p_{1}\left(\mu_{j}\right)=0$ or $p_{2}\left(\mu_{j}\right)=0$ (or both). Applying lemma 3.1.2 (to $p_{1}$ and $-p_{2}$), we have that

1. $p_{1}\left(\mu_{1}\right)=0$ and $p_{1}\left(\mu_{i}\right)<0\forall i\neq 1$ if and only if tr(R)<0.
2. $p_{1}\left(\mu_{k}\right)=0$ and $p_{1}\left(\mu_{i}\right)<0\forall i\neq k$ if and only if tr(R)>0.
3. $p_{2}\left(\mu_{1}\right)=0$ and $p_{2}\left(\mu_{i}\right)>0\forall i\neq 1$ if and only if B>0.
4. $p_{2}\left(\mu_{k}\right)=0$ and $p_{2}\left(\mu_{i}\right)>0\forall i\neq k$ if and only if B<0.
5. $p_{1}\left(\mu_{i}\right)=0$ for all i if and only if tr(R)=0.
6. $p_{2}\left(\mu_{i}\right)=0$ for all i if and only if B=0.

Now, let’s consider some of the possible cases. Notice that by equations (1) and (2) and the continuity of both polynomials with respect to λ, if $p_{1}\left(\mu_{j},\lambda_{0}\right)\neq 0$, then we must have $p_{1}\left(\mu_{j},\lambda_{0}\right)<0$. Similarly, if $p_{2}\left(\mu_{j},\lambda_{0}\right)\neq 0$, then we must have $p_{2}\left(\mu_{j},\lambda_{0}\right)>\;0$.

If $p_{1}\left(\mu_{i}\right)=0$ and $p_{2}\left(\mu_{i}\right)>0$, there are $\alpha_{i}$ complex conjugate pairs critical eigenvalues that are eigenvalues of $Q\left(x_{0},\lambda_{0}\right)+\mu_{i}R\left(x_{0},\lambda_{0}\right)$; if $p_{2}\left(\mu_{i}\right)=0$ and $p_{1}\left(\mu_{i}\right)>0$, then there are $\alpha_{i}$ real critical eigenvalues that are an eigenvalue of $Q\left(x_{0},\lambda_{0}\right)+\mu_{i}R\left(x_{0},\lambda_{0}\right)$; and if $p_{1}\left(\mu_{i}\right)=p_{2}\left(\mu_{i}\right)=0$, there are $2\alpha_{i}$ real critical eigenvalues that are the two eigenvalues of $Q\left(x_{0},\lambda_{0}\right)+\mu_{i}R\left(x_{0},\lambda_{0}\right)$ with multiplicity $\alpha_{i}$.

Lastly, by the Perron-Frobenius Theorem, $\alpha_{k}=1$. We use this information to enumerate all possibilities in Table 1. □

**Theorem 10** (Nondegeneracy Conditions in 2-Dimensions). *Assume that* 𝒩 *has valence* ν. *Suppose that the node space is 2-dimensional and the adjacency matrix* A *has distinct real eigenvalues* $\mu_{1}<\mu_{2}<\ldots <\mu_{k}$ *and corresponding algebraic multiplicities* $\alpha_{1},\alpha_{2},\ldots ,\alpha_{k}$. *Suppose that* $\text{det}\left(R\left(x_{0},\lambda_{0}\right)\right)=0$ *and take*

$$B≔\text{tr}\left(Q\left(x_{0},\lambda_{0}\right)\right)\text{tr}\left(R\left(x_{0},\lambda_{0}\right)\right)-\text{tr}\left(Q\left(x_{0},\lambda_{0}\right)R\left(x_{0},\lambda_{0}\right)\right).$$

1. *Suppose that at* $\left(x_{0},\lambda_{0}\right),(\text{tr}(Q)\neq 0)\vee (\text{tr}(R)\neq 0)$ *where* ∨ *denotes the logical “or.” If*
det(Q)>|νB|
  *then the critical eigenvalues are the pair of imaginary eigenvalues from a single matrix* $Q+\mu_{j}R$, *with multiplicity* $\alpha_{j}$.
2. *Suppose that at* $\left(x_{0},\lambda_{0}\right),(B\neq 0)\vee (\text{det}(Q)\neq 0)$. *If*
$$\text{tr}\left(Q\right)<-\left|\nu \text{tr}\left(R\right)\right|,$$
  *then the critical eigenvalues are a real eigenvalue from a single matrix* $Q+\mu_{j}R$ *with multiplicity* $\alpha_{j}$.

*Proof*. Since we can obtain important information about the eigenvalues of a 2×2 matrix using the trace and determinant, define the following polynomials of μ:

$$p_{1}\left(\mu ,\lambda \right)=\text{tr}(Q\left(x\left(\lambda \right),\lambda \right)+\mu R\left(x\left(\lambda \right),\lambda \right)\;=\text{tr}(\left(Q\left(x\left(\lambda \right),\lambda \right)\right)+\mu \text{tr}\left(R\left(x\left(\lambda \right),\lambda \right)\right)\;p_{2}\left(\mu ,\lambda \right)=\text{det}\left(Q\left(x\left(\lambda \right),\lambda \right)+\mu R\left(x\left(\lambda \right),\lambda \right)\right)$$

Again, this setup is illustrated in Figure 3. We will drop the arguments λ if it is obvious where the polynomial is being evaluated. Since we assume that $\text{det}\left(R\left(x_{0},\lambda_{0}\right)\right)=0$, we have that

$$p_{2}\left(\mu ,\lambda_{0}\right)=[\text{tr}\left(Q\left(x_{0},\lambda_{0}\right)\text{tr}\left(R\left(x_{0},\lambda_{0}\right)\right)-\text{tr}\left(Q\left(x_{0},\lambda_{0}\right)R\left(x_{0},\lambda_{0}\right)\right)\right]\mu +\text{det}\left(Q\left(x_{0},\lambda_{0}\right)\right)\;=\text{det}\left(Q\left(x_{0},\lambda_{0}\right)\right)+\mu B\left(x_{0},\lambda_{0}\right)$$

Suppose that det(Q)>|νB| at the bifurcation. Of course, this implies that det(Q)>0. Furthermore, by the Perron-Frobenius theorem ν is the spectral radius of A (i.e. the largest absolute value of its eigenvalues). Therefore, for any i,

det(Q)>|νB|

$$\Rightarrow \text{det}\left(Q\right)>\left|\mu_{i}B\right|,$$

and

$$p_{2}\left(\mu_{i}\right)=\text{det}(Q)+\mu_{i}B>0.$$

Since $\text{det}\left(Q+\mu_{i}R\right)>0$ for all i, the critical eigenvalues must correspond to pairs of imaginary eigenvalues, given by matrices $Q+\mu_{i}R$ such that $p_{1}\left(\mu_{i}\right)=0$. In the case that tr(Q)≠0, there is exactly one root of the line $p_{1}$, implying that at the bifurcation, there is a unique $\mu_{j}$ such that $p_{1}\left(\mu_{j}\right)=0$. Thus, there is a pair of imaginary critical eigenvalues that are eigenvalues of $Q+\mu_{j}R$. Since $\mu_{j}$ is an eigenvalue of A with multiplicity $\alpha_{j}$, the critical eigenvalue is an eigenvalue of $J≔(dF{)}_{\left(x_{0},\lambda_{0}\right)}$ with multiplicity $\alpha_{j}$. Now, notice that tr(R)≠0 implies tr(Q)<0 (otherwise the synchronous branch would be unstable before the bifurcation), and thus our analysis of the case that tr(R)≠0 reduces to the previous case that tr(Q)≠0.

For (2), suppose that tr(Q)<-|νtr(R)| at the bifurcation. Then tr(Q)<0. Furthermore, for any i,

tr(Q)<−|νtr(R)|

$$\Rightarrow \text{tr}\left(Q\right)<-\left|\mu_{i}\text{tr}\left(R\right)\right|,$$

and

$$p_{1}\left(\mu_{i}\right)=\text{tr}\left(Q\right)+\mu_{i}\text{tr}\left(R\right)<0.$$

Since $p_{1}\left(\mu_{i}\right)<0$ for all i at the bifurcation, any critical eigenvalues must be real and given by matrices $Q+\mu_{i}R$ with $p_{2}\left(\mu_{i}\right)=0$. Using the same logic as previously, if det(Q)≠0, there can be exactly one root of the line $p_{2}$, implying that at the bifurcation there is a unique $\mu_{j}$ with $p_{2}\left(\mu_{j}\right)=0$, giving a real critical eigenvalue that is an eigenvalue of $Q+\mu_{j}R$. Since $\mu_{j}$ is an eigenvalue of A with multiplicity $\mu_{j}$, the critical eigenvalue is an eigenvalue of J with multiplicity $\alpha_{j}$. We also have that B≠0 implies that det(Q)>0 (otherwise the synchronous branch would be unstable before the bifurcation), thus reducing to the prior case. In both cases, since $\text{tr}\left(Q+\mu_{j}R\right)<0$ and $\text{det}\left(Q+\mu_{j}R\right)=0$ the critical eigenvalue is real. □

We have shown that for a 2-dimensional node space, if det(R)=0 – as is the case for Notch signaling (Section 3.2.1) – then any stable pattern that forms generically corresponds to the critical pattern space $P^{\mu_{1}}$. If det(R)≠0, however, then a stable pattern can correspond to any critical pattern space $P^{\mu_{i}}$ as shown below. This indicates two possible mechanisms for the diversity of patterns we see in biological systems: cells reuse chemical signaling pathways but reorganize themselves to change communication, or cells use different chemical signaling pathways.

**Theorem 11** (Existence of Admissible ODE for Arbitrary First Branch, Golubitsky and Stewart). *Suppose the adjacency matrix* A *has distinct real eigenvalues* $\mu_{1}<\ldots <\mu_{k}$. *Then for any* i, *there exists an admissible system with 2-dimensional node space such that the critical pattern space is* $P^{\mu_{i}}$.

*Proof*. See (Golubitsky and Stewart, 2023).

**Theorem 12** (Sufficient Condition for Pattern in $P^{\mu_{1}}$ or $P^{\mu_{k}}$). *Suppose that the node space is 2-dimensional and the adjacency matrix* A *has distinct real eigenvalues* $\mu_{1}<\mu_{2}<\ldots <\mu_{k}$ *not counting multiplicity. If* $\text{det}\left(R\left(x_{0},\lambda_{0}\right)\right)\leq 0$, *then the critical pattern space is either* $P^{\mu_{1}},\;P^{\mu_{k}},\;P^{\mu_{1}}⊕P^{\mu_{k}}$, or $R^{n}$.

*Proof*. As before, let

$$p_{1}\left(\mu ,\lambda \right)=\text{tr}(Q\left(x\left(\lambda \right),\lambda \right)+\mu R\left(x\left(\lambda \right),\lambda \right)\;=\text{tr}(\left(Q\left(x\left(\lambda \right),\lambda \right)\right)+\mu \text{tr}\left(R\left(x\left(\lambda \right),\lambda \right)\right)\;p_{2}\left(\mu ,\lambda \right)=\text{det}\left(Q\left(x\left(\lambda \right),\lambda \right)+\mu R\left(x\left(\lambda \right),\lambda \right)\right).$$

If $p_{1}\left(\cdot ,\lambda_{0}\right)\equiv 0$ or $p_{2}\left(\cdot ,\lambda_{0}\right)\equiv 0$, then there are critical eigenvalues from $Q+\mu_{i}R$ for all i, and the critical pattern space is $R^{n}$.

Now suppose that $p_{1}≢0$ and $p_{2}≢0$. Then, there is a critical eigenvalue that is an eigenvalue of $Q+\mu_{i}R$ if and only if either $p_{1}\left(\mu_{i},\lambda_{0}\right)=0$ or $p_{2}\left(\mu_{i},\lambda_{0}\right)=0$. From Lemma 7.1, if $p_{1}\left(\mu_{i},\lambda_{0}\right)=0$ then i=1,k.

$p_{2}\left(\mu ,\lambda_{0}\right)$ is a quadratic polynomial in μ whose graph is downward opening (Figure 3), so its derivative is linear with slope 2 $\text{det}\left(R\left(x_{0},\lambda_{0}\right)\right)<0$. Suppose that $p_{2}\left(\mu_{i},\lambda_{0}\right)=0$ for i≠1,k for contradiction. We have two cases:

1. If ${\left.\frac{\partial }{\partial \mu }p_{2}\right|}_{\left(\mu_{i},\lambda_{0}\right)}>0$, then $p_{2}\left(\mu_{1},\lambda_{0}\right)<0$ since $\mu_{1}<\mu_{i}$. But this contradicts the continuity of $p_{2}$ in λ since $p_{2}\left(\mu_{i},\lambda_{0}\right)>0$ for $\lambda <\lambda_{0}$, otherwise the branch (x(λ),λ) would not be stable.
2. Similarly, if ${\left.\frac{\partial }{\partial \mu }p_{2}\right|}_{\left(\mu_{i},\lambda_{0}\right)}<0$, then $p_{2}\left(\mu_{k},\lambda_{0}\right)<0$ since $\mu_{k}>\mu_{i}$, which again contradicts the continuity of $p_{2}$ as a function of λ. □

In conclusion, since $p_{1}\left(\mu_{i}\right)=0$ or $p_{2}\left(\mu_{j}\right)=0$ can only occur for i=1,k or j=1,k, there can only be critical eigenvalues from $Q+\mu_{1}R$ or $Q+\mu_{k}R$ (or both), which gives us our conclusion by Proposition 2 and Definition 8.

### 3.2 Predicting patterns from qualitative features of cell-communication and chemical kinetics

We use theorems in Section 3.1 to predict patterns of cell fates given qualitative features of Notch signaling and the cell-communication network.

#### 3.2.1 Critical pattern space of Notch signaling is $P^{\mu_{1}}$

**Fig. 4:**
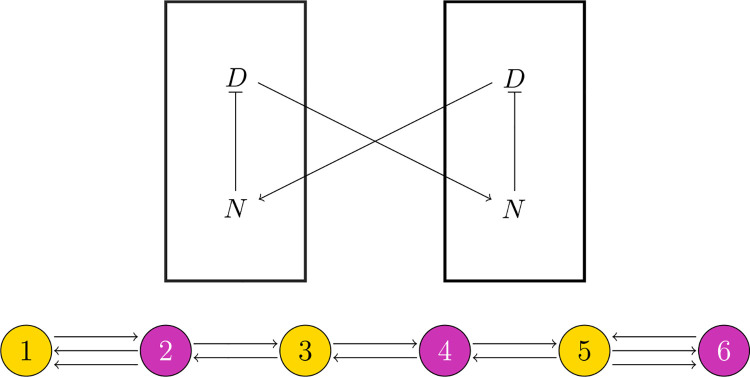
Top: A schematic diagram capturing the known qualitative features of delta-notch chemical signaling. Notch inhibits Delta within a cell, and Delta activates Notch in neighboring cells. Bottom: At one stage in development, the *C. elegans* vulva is a line of six cells. Under a mutation of the let-23 gene, each cell receives approximately the same amount of external signaling, so we can assume that their dynamics are the same; thus, the system can be represented with a regular network. The alternating color is the balanced coloring ⋈ associated with the critical pattern space $P^{\mu_{1}}$ for the Notch pathway

Following the model of Collier et al. (1996), the feedback between Delta and Notch in coupled cells can be represented as in Figure 4 (top) where Notch inhibits Delta within a cell and Delta activates Notch in neighboring cells. It is natural to assume that i *activates* j (represented with i→j) means that

$$\frac{\partial {\dot{x}}_{j}}{\partial x_{i}}>0,$$

and i *inhibits* j (represented with i⊣j) means that

$$\frac{\partial {\dot{x}}_{j}}{\partial x_{i}}<0.$$

Furthermore, we assume that Delta and Notch both decay over time, meaning that

$$\begin{array}{c} \frac{\partial \dot{N}}{\partial N}<0 \end{array}$$

$$\frac{\partial \dot{D}}{\partial D}<0$$

Writing the concentrations of the biochemical species as a vector (D,N), the internal and coupled dynamics of the Delta-Notch signaling pathway take the form

$$\begin{array}{ll} Q=\left(\begin{array}{cc} - & -\\ 0 & - \end{array}\right) & R=\left(\begin{array}{cc} 0 & 0\\ + & 0 \end{array}\right) \end{array}$$

where + denotes a positive term, and – denotes a negative term. Thus, we have det(R)=0; if A has real eigenvalues, we can apply Theorem 9.

We also have that

$$\begin{array}{ll} B=\text{tr}\left(Q\right)\text{tr}\left(R\right)-\text{tr}\left(QR\right)>0 & \\ \text{tr}\left(Q\right)<0=-\left|\nu \text{tr}\left(R\right)\right| & (\text{NDG}\;\text{condition},\;\text{Theorem}\;10), \end{array}$$

so from Table 1, the critical pattern space is $P^{\mu_{1}}$.

#### 3.2.2 Preferred pattern in mutated *C. elegans* vulval precursor cells (VPCs)

The *C. elegans* vulval precursor cells (VPCs) form a pattern that is mediated by Notch signaling. With a mutation of let-23, all VPCs receive approximately equal external signals (Aroian and Sternberg, 1991), so the system can be represented with the regular network in Figure 4 (bottom). The adjacency matrix is

$$A=\left(\begin{array}{llllll} 0 & 2 & 0 & 0 & 0 & 0\\ 1 & 0 & 1 & 0 & 0 & 0\\ 0 & 1 & 0 & 1 & 0 & 0\\ 0 & 0 & 1 & 0 & 1 & 0\\ 0 & 0 & 0 & 1 & 0 & 1\\ 0 & 0 & 0 & 0 & 2 & 0 \end{array}\right).$$

Using Matlab, we find that the eigenvalues are real, and the smallest eigenvalue and its corresponding eigenvector are

$$\begin{array}{ll} \mu_{1}=-2 & v_{1}=\left(1,-1,1,-1,1,-1\right), \end{array}$$

implying that the critical pattern space $P^{\mu_{1}}={\text{span}}_{R}\left\{v_{1}\right\}.\mu_{1}$ has geometric multiplicity 1, so the critical eigenspace is 1-dimensional (Table 1), and the critical pattern space $P^{\mu_{1}}$ corresponds to the balanced coloring ⋈ shown in Figure 4 (bottom), so Theorem 3 implies that there is a bifurcating branch of solutions with the pattern ⋈. Furthermore, Proposition 4 implies that this will generically be the only stable branch, so we expect the *C. elegans* vulva, with a mutation of let-23, to exhibit an alternating pattern of cell fates – and this is exactly what is observed experimentally.

#### 3.2.3 Preferred patterns in square arrays of cells developing according to Notch signaling

**Fig. 5:**
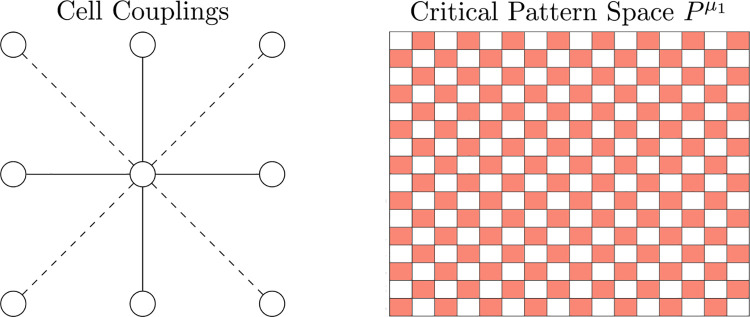
We consider a 16 × 16 array of cells that develops according to the Notch pathway with nearest and next nearest neighbor couplings of cells, as shown on the left. Our theory predicts that the cells will form the checkerboard pattern on the right, which matches our simulations

**Fig. 6:**
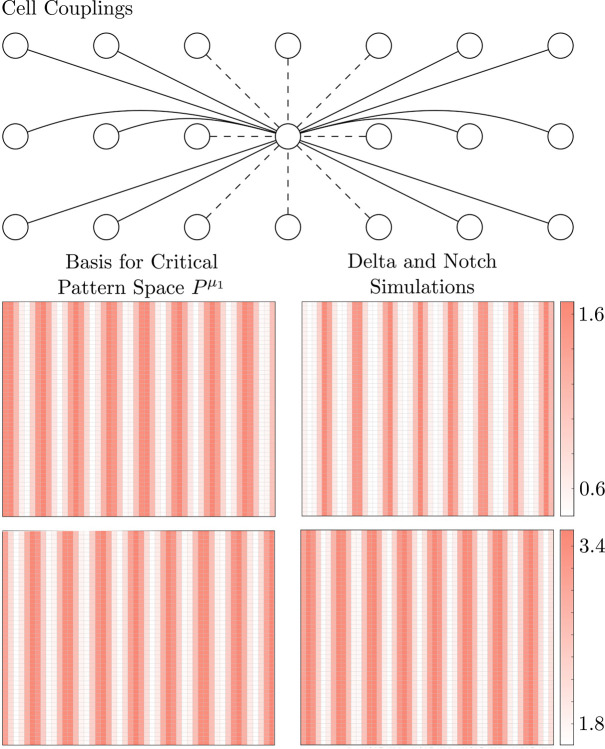
Now we consider a 50 × 50 array of cells that can communicate over long ranges as shown on the top. The critical pattern space is the 2-dimensional space spanned by the vectors on the left. Since the critical pattern space is 2-dimensional, a bifurcation is not guaranteed; however, our simulations of Delta and Notch (top right and bottom right, respectively) show that the pattern of cell fates is in the critical eigenspace, demonstrating that the theory still has merit in more degenerate cases

We will compute the critical pattern space of two square arrays of cells that develop according to Notch signaling (Figures 5,6), showing that cells can change their communication to form a different pattern despite using the same signaling mechanism.

We have shown that the critical pattern space for the Notch pathway is $P^{\mu_{1}}$, so we must compute $P^{\mu_{1}}$ to determine the preferred pattern of the tissue. $P^{\mu_{1}}$ depends on the cell-communication network. In Figure 5, we consider a 16 × 16 array of cells, where each cell is coupled to its neighbors as depicted on the left (for clarity, we omit arrows pointing towards the central cell), and there are periodic boundary conditions. Dotted lines represent a connection strength of 1, and solid lines represent a connection strength of 3. Since there are periodic boundary conditions, the adjacency matrix A will be real and symmetric, so A will have real eigenvalues. Using Matlab, we find that the smallest eigenvalue $\mu_{1}$ gives the checkerboard pattern on the top-right, which matches our simulations.

In Figure 6, we consider a 50 × 50 array of cells with couplings shown in the top panel and periodic boundary conditions. Using Matlab, the minimal eigenvalue $\mu_{1}$ has geometric multiplicity 2, and the critical pattern space is the 2-dimensional space spanned by the vectors depicted on the left of Figure 6. In Table 1, $\beta_{1}=2$, and we do not attempt to show that $\text{dim}\left(E^{\mu }\cap \Delta_{⋈}\right)=1$ for some coloring ⋈, so the hypotheses of Theorem 3 may fail. Thus, we cannot ensure that there is a solution with the pattern of the critical pattern space; however, our numerical simulations validate that the predicted pattern is realized for some parameter range, as shown on the right of Figure 6.

### 3.3 Inferring biochemical interactions from observed patterns

Finally, we show how the theory can be used to infer properties of the chemical signaling pathway from an observed pattern.

**Fig. 7:**
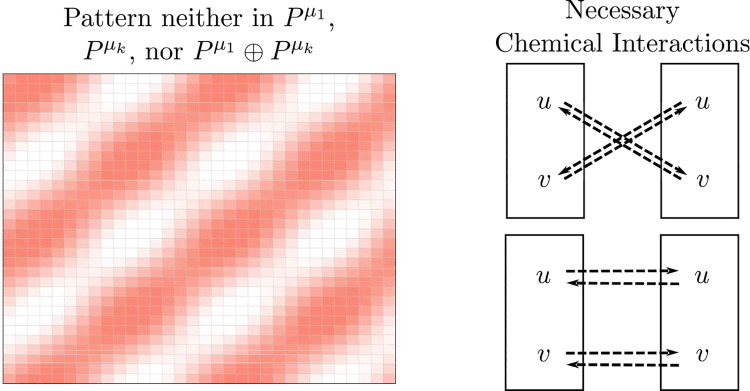
Assuming that the cells are coupled as in figure 5 (left), the given pattern is neither $P^{\mu_{1}},\;P^{\mu_{k}}$, nor $P^{\mu_{1}}⊕P^{\mu_{k}}$. Barring the degenerate case that the critical pattern space is $R^{n}$, by Theorem 12 we must have that det(R)>0, so the development is generically due to a chemical signaling network with either of the connections on the right

For a 16 × 16 array of cells communicating as in Figure 5 (left), if we observe the striped pattern in Figure 7 (left), then the critical pattern is neither $P^{\mu_{1}},\;P^{\mu_{k}}$, nor $P^{\mu_{1}}⊕P^{\mu_{k}}$. Barring the extremely degenerate case that the critical pattern space is $R^{n}$, we must have that det(R)>0 (Theorem 12), so the development is due to a chemical signaling network with the connections given on the right of Figure 7 (top or bottom).

Now, suppose there is some regular array of uniform cells. If we have partial information about chemical signaling in a tissue, we can use the observed tissue pattern to obtain additional information about chemical signaling by using the following steps.

1. Use the partial information about chemical interactions to create matrices representing the internal dynamics Q and coupled dynamics R with variables as entries.
2. Use Theorems 6 and 7 to determine the signs of several variables in Q and R.
3. Check det(R)=0. Then reference the observed pattern against Table 1 to determine necessary conditions on B and R.
4. Use newfound information about B and tr(R) to determine the possible signs of additional variables in Q and R.
5. Lastly, convert the information about the sign of entries in Q and R to information about activation and inhibition of biochemical species.

**Fig. 8:**
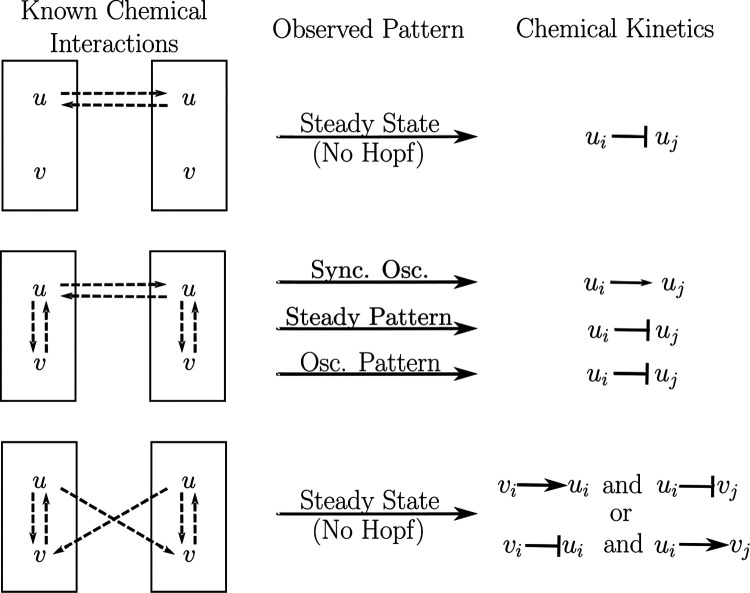
In section 3.3, we infer qualitative features of chemical kinetics given partial information about the chemical signaling network and the type of pattern that forms in a tissue. The analysis is summarized above. “Sync. osc.” is short “synchronous oscillations” and “osc. pattern” is short for “oscillating pattern”

For the first example, consider when one chemical u in a cell influences the same chemical in a neighboring cell, and suppose that the chemical is independent of other chemical species. Then the node space is 1-dimensional, and we can represent the internal and coupled dynamics as in Figure 8 (top). This tells us that

$$Q=\frac{\partial {\dot{u}}_{i}}{\partial u_{i}}$$

$$R=\frac{\partial {\dot{u}}_{i}}{\partial u_{j}}.$$

For the uniform or synchronous state to be stable, we must have that Q<0 (Proposition 6), implying that u decays. Assume that u decays at the same rate for all time. Then for a pattern to form, there must be a synchrony-breaking bifurcation, which by Theorem 8 occurs if and only if R<0, meaning that u in one cell must inhibit u in its neighboring cells, and the inhibition must increase for a synchrony-breaking bifurcation to occur.

Now, suppose we know that the concentration of u is affected by the concentration of another chemical v within the cell (Figure 8, middle). Then writing the chemical species as a vector (u,v),

$$Q=\left(\begin{array}{ll} a & b\\ c & d \end{array}\right)$$

$$R=\left(\begin{array}{ll} \kappa & 0\\ 0 & 0 \end{array}\right)$$

for some real numbers a,b,c,d,κ.

If the cells are initially uniform, then from Proposition 7,

tr(Q)=a+d<0.

This is satisfied if both chemical species decay – as is standard. Furthermore, notice that

det(R)=0,

so we can apply Theorem 9. If the cells oscillate in sync, then we may assume that there was a synchrony-preserving Hopf bifurcation, implying that tr(R)=κ>0 (Table 1, row 4), so $u_{i}\to u_{j}$. If they oscillate in a pattern, then we may assume there was a synchrony-breaking Hopf bifurcation, implying that tr(R)=κ<0 (Table 1, row 2) and thus $u_{i}⊣u_{j}$. If we see that a steady-state pattern has formed among the cells, then we can infer that a synchrony-breaking steady-state bifurcation occurred (Table 1, row 1) meaning that

B=tr(Q)tr(R)−tr(QR)=(a+d)κ−aκ=dκ>0.

Assuming that v decays, d<0, implying that $u_{i}⊣u_{j}$ and the strength of decay of v or the strength of inhibition must increase for a pattern to form.

Lastly, assume that u in cell i influences v in neighboring cells j as shown by the general diagram in Figure 8 (bottom). Writing the chemical species as a vector (u,v), we have that the internal and coupled dynamics are given by

$$Q=\left(\begin{array}{ll} a & b\\ c & d \end{array}\right)$$

$$R=\left(\begin{array}{ll} 0 & 0\\ \kappa & 0 \end{array}\right)$$

for real numbers a,b,c,d,κ.

If the cells are initially uniform, then from Proposition 7,

tr(Q)=a+d<0.

Again, this is satisfied if both chemical species decay. Furthermore, notice that

$$\begin{array}{ll} \text{det}\left(R\right)=0 & \\ \;\text{tr}\left(Q\right)<-\left|\nu \text{tr}\left(R\right)\right|=0 & \left(\text{NDG}\;\text{condition},\;\text{Theorem}\;10\right)\text{,} \end{array}$$

so applying Theorem 9, we are in the first or third row of Table 1. This means there can only be a steady-state bifurcation (i.e. no oscillatory behavior). And

B=tr(Q)tr(R)-tr(QR)=-bκ.

If sgn(b)=sgn(κ), then synchrony cannot be broken since B<0 (see Table 1). If we observe a pattern, however, then we expect that sgn(b)≠sgn(κ), meaning that we either have

1. $v_{i}\to u_{i}$ and $u_{i}⊣v_{j}$
2. or $v_{i}⊣u_{i}$ and $u_{i}\to v_{j}$.

## 4 Discussion

This framework provides powerful tools for molecular biologists who are interested in uncovering the mechanisms of pattern formation. It provides a systematic approach to identifying the molecular causes of pattern failures, which can help guide protein knockdown experiments in model organisms. Additionally, our theory can help uncover unknown molecular interactions in chemical signaling networks through the analysis of patterns that emerge in tissues (Figure 8).

This framework was created by recognizing that a developing tissue can be modeled as a system of ODEs on a regular network. When the linearization has null eigenvalues, a pattern can form through a bifurcation (Theorem 3). Using the network framework, we can determine the eigenvalues by examining smaller matrices $Q+\mu_{i}R$, where $\mu_{i}$ represents an eigenvalue of the adjacency matrix A, while Q and R represent the linearized internal and coupled dynamics, respectively (Proposition 2). Thus, the pattern is determined by both the global cell-communication structure $\left(\mu_{i}\right)$ and the local cell-level dynamics (Q and R).

We use our assumptions about a developing tissue to gain information about the pattern and chemical kinetics. Since the synchronous state is initially stable, all eigenvalues are negative, which provides crucial information about the chemical kinetics described by Q and R (Propositions 6, 7). Then for a pattern to form, $Q+\mu_{i}R$ must have a null eigenvalue for some $\mu_{i}$. We found that in nondegenerate systems with either one or two biochemicals and one coupling between cells, the resulting steady-state pattern always corresponds to the smallest eigenvalue of A (denoted $\mu_{1}$) and is given by the critical pattern space $P^{\mu_{1}}$ (Definition 8). The formation of the pattern, however, requires specific conditions on Q and R (Theorems 8, 9).

Altogether, our theory suggests that (1) if the chemical kinetics Q and R satisfy certain conditions, then a pattern $P^{\mu_{1}}$ will form that is dictated by the cell-communication structure A (Section 3.2); and (2) if a pattern $P^{\mu_{i}}$ forms in an array with known communication structure A, then the chemical kinetics Q and R must satisfy certain properties (Section 3.3).

Our framework also extends beyond basic research with several potential applications. In tissue engineering, our findings suggest that controlling the range of cell-communication can guide pattern formation (Section 3.2.3). Alternatively, maintaining the same communication structure while adjusting chemical signaling can alter the tissue pattern (Figure 7). In medicine, these insights have important implications for understanding disease mechanisms, as disruptions in pattern formation are associated with congenital malformations. We provide a theoretical framework for understanding the molecular changes underlying these failures. In particular, our theory illustrates that multiple molecular factors can lead to the same pattern breakdown. This helps explain why drugs can treat a disease for some individuals but not others and suggests novel drug targets.

Although our framework relies on simplified assumptions about biological systems, future research will refine and expand its applicability. A key next step will be extending our analysis from two-dimensional signaling networks to more complex, realistic biochemical systems. This expansion will improve our ability to identify the molecular causes of pattern formation. Additionally, we plan to validate our theoretical predictions through simulations that incorporate spatial features including morphogen gradients, parameter noise, and network perturbations, enabling us to apply our theory to a broader range of biological contexts.

## Figures and Tables

**Table 1:** Enumeration of all critical pattern spaces, the multiplicity and type of critical eigenvalues, and the dimension of the critical eigenspaces, given certain conditions on the trace and the determinant of the internal and coupled dynamics. The condition “NDG” stands for non-degeneracy and refers to some condition on the trace or determinant of $Q+\mu_{i}R$ that ensures it is nonzero for every i. An example of such conditions is given in Theorem 10. If $\beta_{1}=1$, the first four bifurcations have simple critical eigenvalues and thus generically lead to a steady-state or Hopf bifurcations with critical pattern spaces $P^{\mu_{1}}$ (synchrony-breaking) or $P^{\mu_{k}}$ (synchrony-preserving).

Critical Pattern Space	Critical Eigenvalues	Dimension of Critical Eigenspace	Determinant Condition	Trace Condition
$P^{\mu_{1}}$	$\alpha_{1}$ (real)	$\beta_{1}$	B>0	NDG
$P^{\mu_{1}}$	$2\alpha_{1}$ (imag.)	$\beta_{1}$	NDG	tr(R)<0
$P^{\mu_{k}}$	1 (real)	1	B<0	NDG
$P^{\mu_{k}}$	2 (imag.)	1	NDG	tr(R)>0
$P^{\mu_{1}}$	$2\alpha_{1}$ (real)	$2\beta_{1}$	B>0	tr(R)<0
$P^{\mu_{k}}$	2 (real)	2	B<0	tr(R)>0
$P^{\mu_{1}}⊕P^{\mu_{k}}$	$\alpha_{1}$ (real) 2 (imag.)	$\beta_{1}+1$	B>0	tr(R)>0
$P^{\mu_{1}}⊕P^{\mu_{k}}$	$2\alpha_{1}$ (imag.) 1 (real)	$\beta_{1}+1$	B<0	tr(R)<0
$R^{n}$	$2\alpha_{1}$ (real) $\sum_{j\neq 1}2\alpha_{j}$ (imag.)	$n+\beta_{1}$	B>0	tr(R)=0
$R^{n}$	2 (real) $\sum_{j\neq k}2\alpha_{j}$ (imag.)	n+1	B<0	tr(R)=0
$R^{n}$	$2\alpha_{1}$ (real) $\sum_{j\neq 1}\alpha_{j}$ (real)	$n+\beta_{1}$	B=0	tr(R)<0
$R^{n}$	2 (real) $\sum_{j\neq k}\alpha_{j}$ (real)	n+1	B=0	tr(R)>0
$R^{n}$	$\sum_{1\leq j\leq k}2\alpha_{j}$ (imag.)	n	NDG	tr(R)=0
$R^{n}$	$\sum_{1\leq j\leq k}\alpha_{j}$ (real)	n	B=0	NDG
$R^{n}$	$\sum_{1\leq j\leq k}2\alpha_{j}$ (real)	2n	B=0	tr(R)=0

## Data Availability

The code referenced in this paper is available on Github at https://github.com/ldobrien1234/cell-pattern-validation. It contains the Matlab code used to find the critical pattern space in a square array of cells and simulations that verify our theoretical predictions as shown in Figures 5,6. All figures were created using Inkscape and TikZ.
